# Supplementary figures and images for: Long non-coding RNAs (lncRNAs) NEAT1 and MALAT1 are differentially expressed in severe COVID-19 patients: An integrated single-cell analysis
Source: PLoS One. 2022 Jan 10;17(1):e0261242. doi: 10.1371/journal.pone.0261242 (PMC8746747; doi:10.1371/journal.pone.0261242)

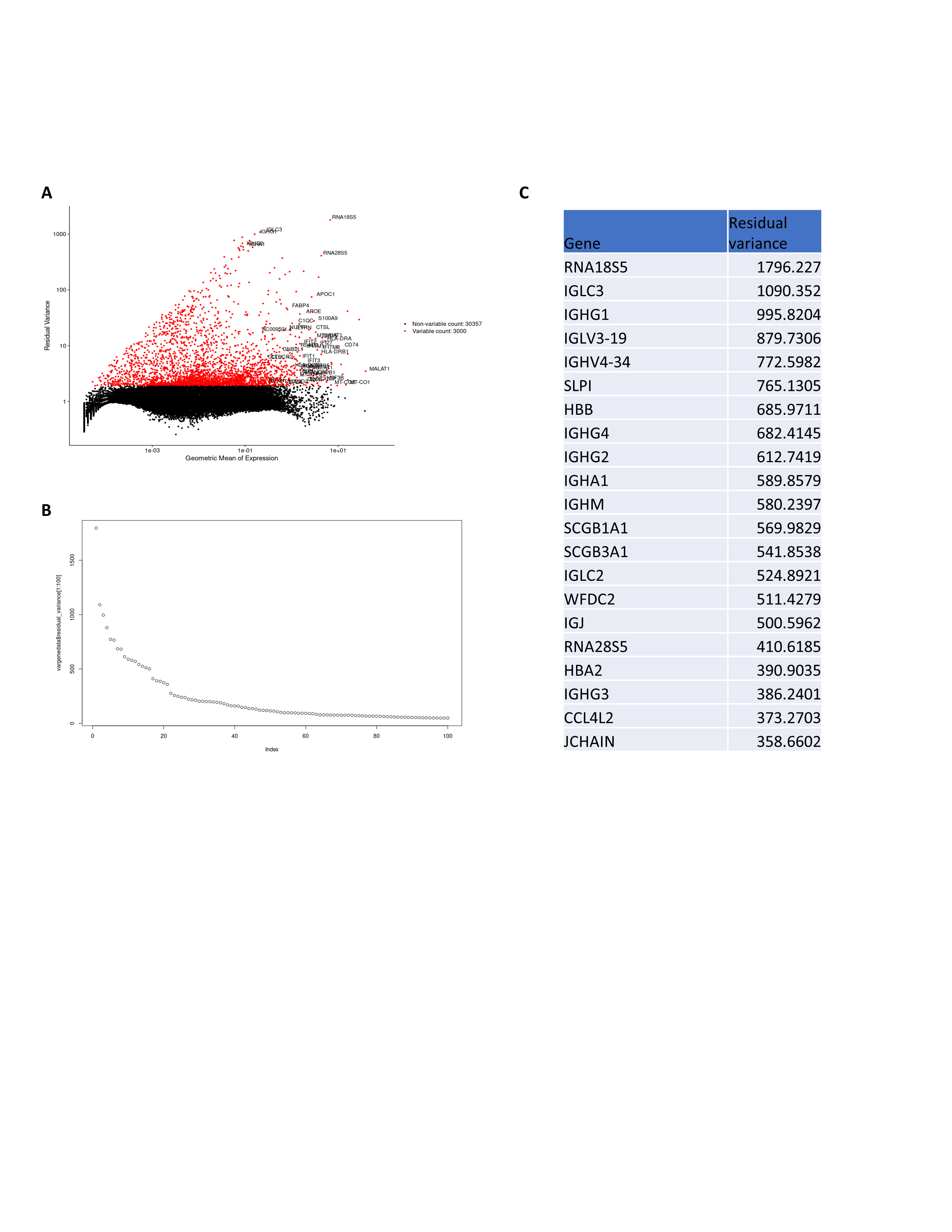

Supplement: S1 Fig — A. A plot of mean expression levels versus residual variance for all genes detected in dataset. Genes with the highest residual variance are nearly all immunoglobulin and ribosome genes. B. Plot of 100 genes with highest residual variance in descending order. The rate of variance decrease stabilizes after the 21st gene. C. Table of top 21 genes filtered out of downstream analysis due to very high residual variance. (BMP) [file pone.0261242.s001.bmp]

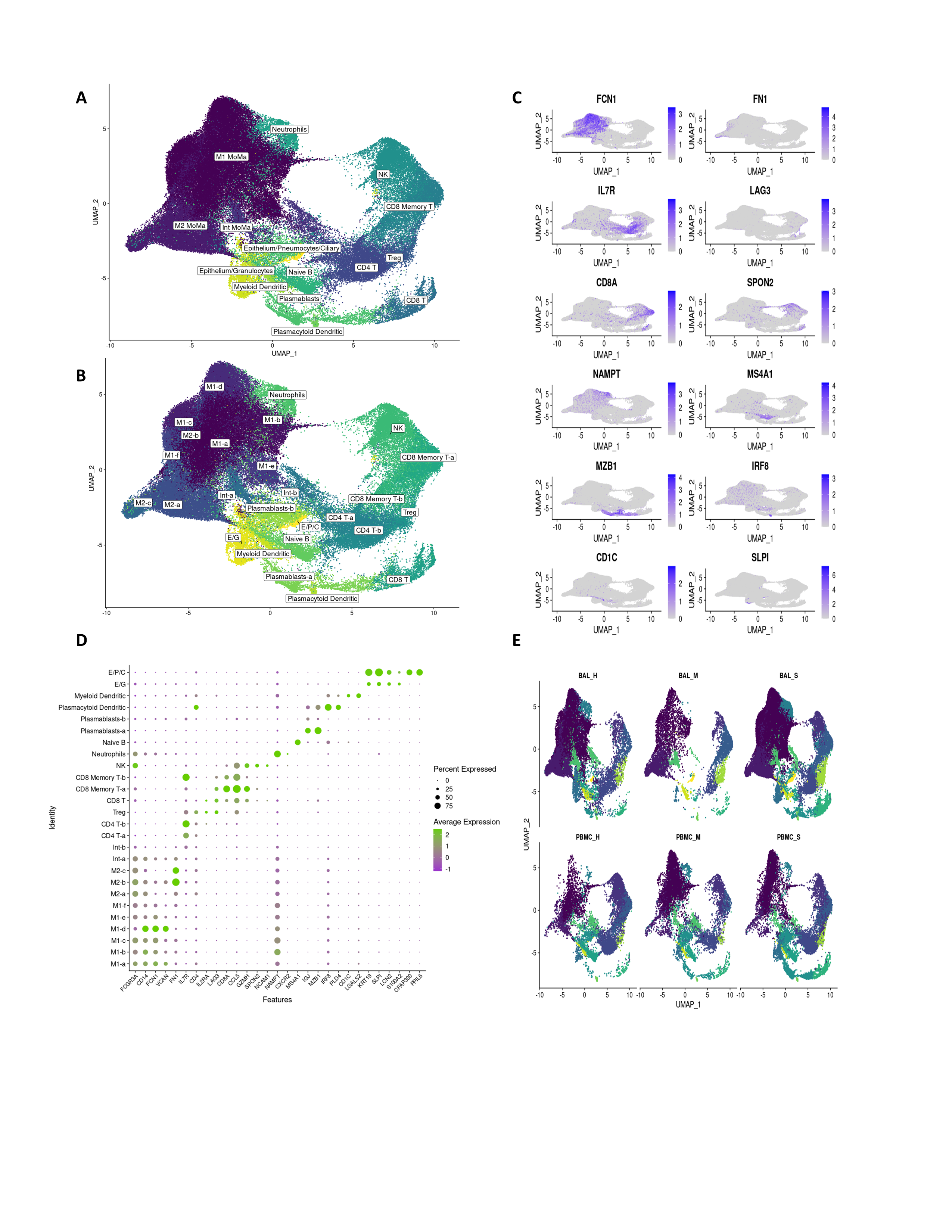

Supplement: S2 Fig — A/B. Uniform manifold approximation and projection (UMAP p)lots showing the distribution of all cells in both cohorts within defined cell types. Plot A shows the 15 major cell groups we identified with labels over the cluster centers. Plot B shows the subclusters making up those groups. The major groups are abbreviated as prefixes with a letter suffix indicating subgroup. M1, M2 and Int are the macrophage/monocytes. NK is natural killer cells, E/G is epithelial cells and granulocytes, and E/P/C is epithelia, pneumocyte, and ciliary cells. C. Select markers for major cell groups plotted on the same UMAP projection. This illustrates the specificity of these markers for different regions of the plot corresponding to our respective cell types. D. Dot plot visualization of top markers utilized for identification of each cell cluster. The size of each dot indicates the percentage of cells with detectable expression of each gene, with color indicating expression level. E. Splitting the UMAP by cohort and by severity, with “H” indicating healthy control, “M” indicating mild disease, and “S” indicating severe disease illustrates that cell clusters do not organize according to sample type or patient condition, indicating successful integration of the datasets. (BMP) [file pone.0261242.s002.bmp]

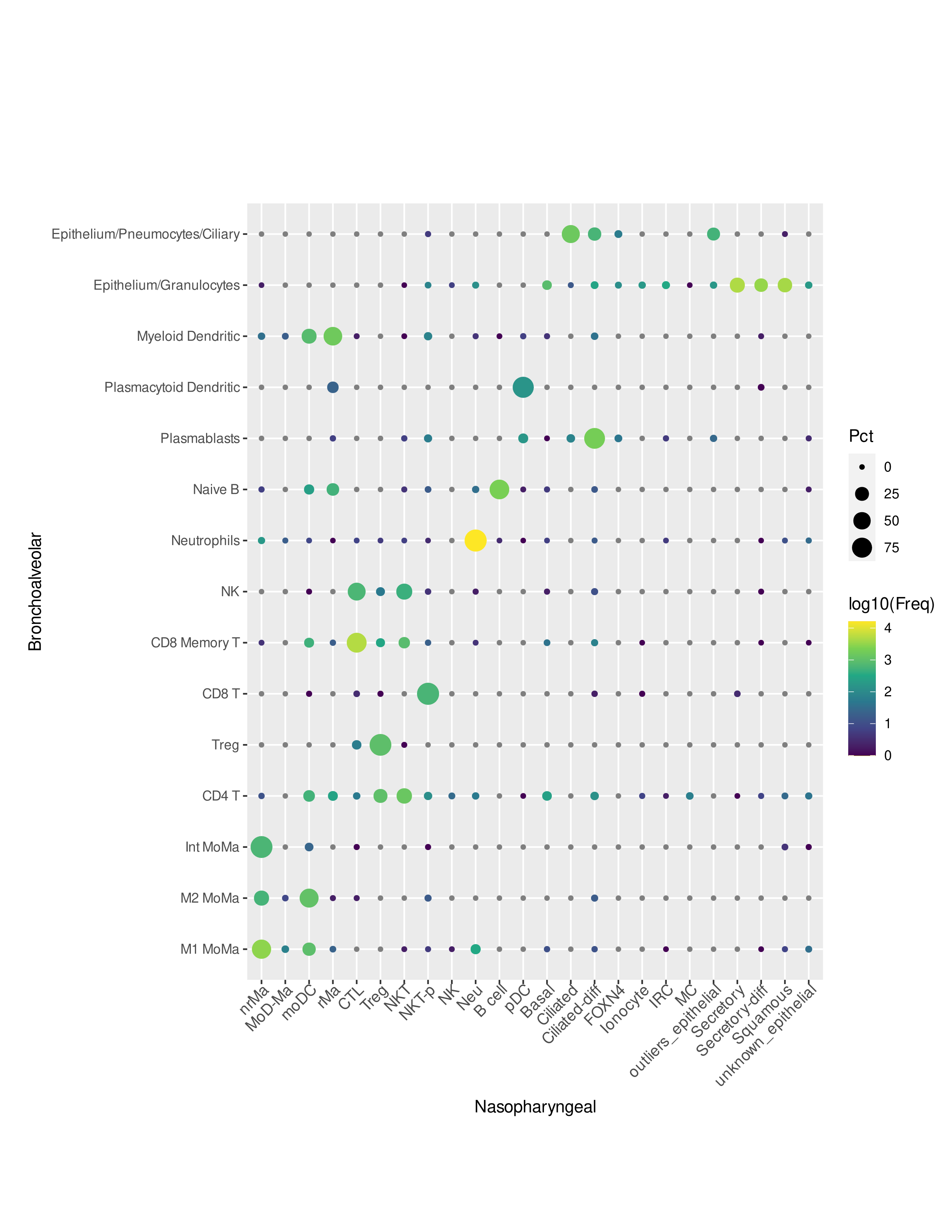

Supplement: S3 Fig — Dot plot shows cells from the nasopharyngeal validation dataset, identified by their transferred labels on the Y axis and their original labels on the X axis. Color of each dot indicates the numeric log frequency of cells that fit each corresponding set of labels. The size of each dot represents the percentage of each transferred cell types which is represented by each original cell type. From left to right, the full cell type names from the nasopharyngeal dataset are: non-resident macrophage (nrMa), monocyte-derived macrophage (MoD-Ma), monocyte-derived dendritic cell (moDC), resident macrophage (rMa), cytotoxic T cell (CTL), regulatory T cell (Treg), natural killer T cell (NKT), proliferating natural killer T cell (NKT-p), natural killer cells (NK), neutrophils (Neu), B cells, plasmacytoid dendritic cell (pDC), basal cell, ciliated cell, differentiating ciliated cell, FOXN4+ epithelial cell, ionocyte, interferon responsive cell (IRC), mast cell (MC), epithelial outliers, secretory, differentiating secretory, squamous, and unknown epithelial. (BMP) [file pone.0261242.s003.bmp]

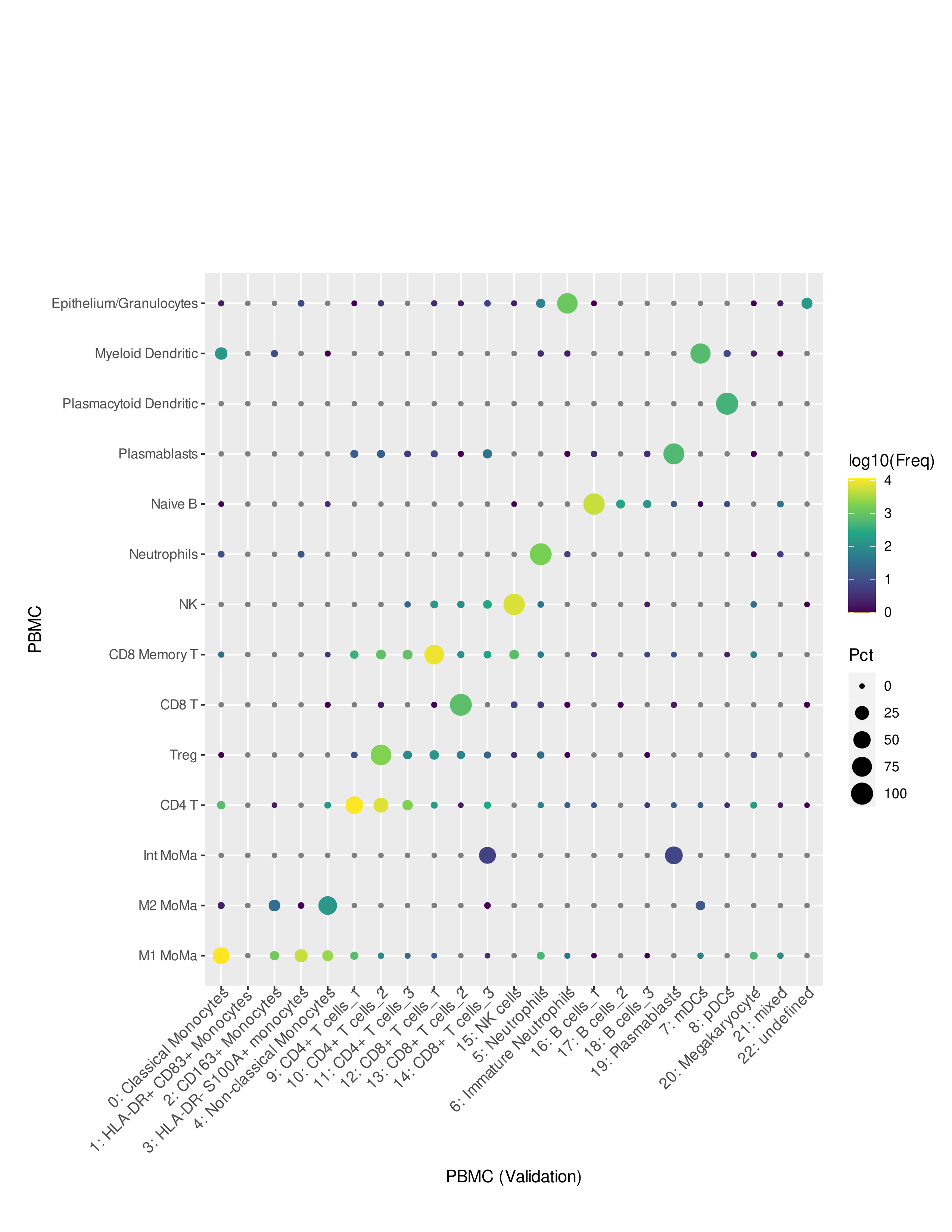

Supplement: S4 Fig — Dot plot shows cells from the PBMC validation set, identified by their transferred labels on the Y axis and their original labels on the X axis. Color of each dot indicates the numeric log frequency of cells that fit each corresponding set of labels. The size of each dot represents the percentage of each transferred cell type represented by each original cell type. This dataset contained several cell types where the same label was applied to more than one subcluster, resulting in numeric suffixes for similar cell types. mDCs correspond to myeloid dendritic cells and pDCs correspond to plasmacytoid dendritic cells. (BMP) [file pone.0261242.s004.bmp]
