# Supplementary material for: Long non-coding RNAs (lncRNAs) NEAT1 and MALAT1 are differentially expressed in severe COVID-19 patients: An integrated single-cell analysis
Source: PLoS One. 2022 Jan 10;17(1):e0261242. doi: 10.1371/journal.pone.0261242 (PMC8746747; doi:10.1371/journal.pone.0261242)
Supplement: S1 Table — Patient information from the BAL and PBMC cohorts used in this analysis. Patients who were intubated or had PaO2/FiO2 ≤ 300 mmHg were classified as severe. In BAL, patient “Mild 3” had only 369 cells recovered after filtering and was only used for initial clustering. Exact ages were not available in the PBMC cohort. The first patient in this cohort was sampled twice, once while classified as a mild patient, and once after their symptoms worsened and required mechanical ventilation. Several patients in the PBMC cohort received azithromycin, which can have immunomodulatory effects before sample collection. (PDF) [file pone.0261242.s005.pdf]

Table S1: Key characteristics of patients within each dataset. Patient information from the BAL and PBMC cohorts used in this analysis. Patients who were intubated or had PaO2/FiO2 ≤ 300 mmHg were classified as severe. In BAL, patient "Mild 3" had only 369 cells recovered after filtering and was only used for initial clustering. Most patients in the BAL cohort. Exact ages were not available in the PBMC cohort. The first patient in this cohort was sampled twice, once while classified as a mild patient, and once after their symptoms worsened and required mechanical ventilation. Several patients in the PBMC cohort received azithromycin, which can have immunomodulatory effects before sample collection.

Subject	Cohort	Age	Gender	Sample Time (Days After Onset)	Interferon	Ribavirin	Methylprednisone	Azithromycin	Outcome
Mild 1	BAL	36	male	11	yes	yes	no	-	Discharged
Mild 2	BAL	37	female	9	no	no	no	-	Discharged
Mild 3	BAL	35	male	13	yes	yes	no	-	Discharged
Severe 1	BAL	62	male	11	yes	yes	no	-	Discharged
Severe 2	BAL	66	male	18	yes	yes	yes	-	Deceased
Severe 3	BAL	63	male	14	yes	yes	yes	-	Deceased
Severe 4	BAL	65	female	15	yes	yes	yes	-	Discharged
Severe 5	BAL	57	female	8	yes	no	yes	-	Discharged
Severe 6	BAL	46	male	11	yes	yes	no	-	Discharged
Mild 1	PBMC	60-69	male	9	-	-	-	yes	Discharged
Severe 1				11					
Mild 2	PBMC	40-49	male	16	-	-	-	no	Discharged
Mild 3	PBMC	50-59	male	15	-	-	-	no	Discharged
Mild 4	PBMC	20-29	male	12	-	-	-	no	Discharged
Severe 2	PBMC	30-39	male	9	-	-	-	yes	Discharged
Severe 3	PBMC	30-39	male	9	-	-	-	yes	Discharged
Severe 4	PBMC	>80	male	2	-	-	-	no	Deceased
