# Supplementary material for: Long non-coding RNAs (lncRNAs) NEAT1 and MALAT1 are differentially expressed in severe COVID-19 patients: An integrated single-cell analysis
Source: PLoS One. 2022 Jan 10;17(1):e0261242. doi: 10.1371/journal.pone.0261242 (PMC8746747; doi:10.1371/journal.pone.0261242)
Supplement: S2 Table — All healthy controls used from both the BAL and PBMC cohorts are listed. (PDF) [file pone.0261242.s006.pdf]

Table S2: Demographic characteristics of healthy subjects. All healthy controls used from both the BAL and PBMC cohorts are listed.

Subject	Cohort	Age	Gender
Healthy control 1	BAL	38	female
Healthy control 2	BAL	24	male
Healthy control 3	BAL	22	male
Healthy control 1	PBMC	49	female
Healthy control 2	PBMC	49	male
Healthy control 3	PBMC	36	female
Healthy control 4	PBMC	49	male
Healthy control 5	PBMC	48	male
Healthy control 6	PBMC	37	male
