# Supplementary material for: Long non-coding RNAs (lncRNAs) NEAT1 and MALAT1 are differentially expressed in severe COVID-19 patients: An integrated single-cell analysis
Source: PLoS One. 2022 Jan 10;17(1):e0261242. doi: 10.1371/journal.pone.0261242 (PMC8746747; doi:10.1371/journal.pone.0261242)
Supplement: S1 Methods — (DOCX) [file pone.0261242.s016.docx]

**Supplemental Methods:**

*Analysis System Setup:*

All analyses were completed on the same computer running Ubuntu 20.04 with 128GB of RAM and a 16-core processor. Processor core utilization was monitored and tweaked using the R package *future* to optimize performance. Full system and package information is available from the *session.info()* is provided in **S1 File**.

*R packages utilized in analysis:*

The following R packages were directly utilized in the analysis of this manuscript and creation of its figures:

Seurat 3.2.2, GEOquery 2.56.0, tidyverse 1.3.0, SeuratDisk 0.0.0.9010, ggplot2 3.3.2, sctransform 0.3, future 1.19.1, MAST 1.14.0, qs 0.23.2, EnhancedVolcano 1.6.0, xlsx 0.6.4.2, gridExtra 2.3, monocle3 0.2.2, topGO 2.40.0 , ComplexHeatmap 2.4.3, ggpubr 0.4.0, circlize 0.4.10, RColorBrewer 1.1-2, ggrepel 0.8.2, dplyr 1.0.2

## Dataset preprocessing and integration:

For BAL, raw count matrices were downloaded from GSE145926. The metadata was extracted from the corresponding series matrix file using the getGEO function in R. Patient identifications were extracted from this metadata for downstream processing. Additional metadata was downloaded from the study’s corresponding github page (<https://github.com/zhangzlab/covid_balf>). This included numbered and codified patient severity categories which were incorporated into the metadata from getGEO. Next, the raw count matrices were imported into R as Seurat objects using the Read10X_h5 function. Each imported sample was matched to its metadata and the metadata was imported into the corresponding Seurat object.

For PBMC, raw count matrices already in Seurat object format were downloaded from the COVID-19 Cell Atlas (https:/www.covid19cellatlas.org/#wilk20). This data was imported directly to R as a Seurat object using the readRDS function. Since this data object already contained all necessary metadata, no additional metadata was downloaded for this dataset.

All samples were then preprocessed according to Seurat’s recommended workflow. First, each dataset was scanned using the PercentageFeatureSet function for reads whose names start with “MT-“, which indicates mitochondrial transcripts. The percentage of all reads for each cell classified as mitochondrial is saved to the dataset’s metadata as an indicator of cellular stress. Next, each dataset is filtered via the subset function with the following settings: nFeature_RNA > 200 & nFeature_RNA < 6000 & percent.mt < 10 & nCount_RNA > 1000. This removes any cells with fewer than 200 genes or more than 6000 genes detected, cells with 10% or more transcripts identified as mitochondrial, and cells with fewer than 1000 total transcripts detected.

Each dataset is then normalized using the sctransform algorithm via the function SCTransform (SCT), with the percentage of mitochondrial transcripts set as a variable to regress. All datasets are then combined into a list to facilitate integration. First, the function SelectIntegrationFeatures is used to find 3000 integration features. Then integration preprocessing is performed via PrepSCTIntegration. Next, the function FindIntegrationAnchors is used to calculate anchors from the first 50 principle components of the dataset. Finally, the data is integrated using the IntegrateData function. After integration, the patient codes from each study were normalized to facilitate comparison. Using the criteria dictated in our methods section, we assigned severity to each patient, changing patients designated as “critical” in the BAL dataset to “severe” since the PBMC dataset did not distinguish between the two groups. Additionally, PBMC and BAL prefixes were added to the corresponding samples’ metadata to facilitate subsequent analysis of each group.

## *Clustering and Identification*

The integrated dataset was further processed by first running principal component analysis (PCA) dimension reduction with default settings using Seurat’s RunPCA function. Then, the FindNeighbors function was used with the first 30 principal components to generate a nearest neighbor graph that facilitates clustering. Louvain clustering was performed using the FindClusters function with a resolution setting of 0.5. Subsequently, a dimension-reduced visualization of this clustering output was generated using the RunUMAP function with 30 principal components to perform uniform manifold approximation and projection (UMAP).

The raw integrated data is then selected with the DefaultAssay function, and SCT is once again applied with the same settings as in preprocessing. This time, the algorithm is normalizing the entire integrated dataset instead of each sample. Once this was completed, the normalized and integrated data can be used for differential analysis including finding markers for each cell cluster for identification and finding differentially expressed genes (DEGs) for downstream analysis.

Cell identification was performed in a multistep process. First, an initial identity was assigned to each cluster based on manual analysis of unique gene markers for each cluster. Next, a higher resolution clustering step was used to generate additional subclusters and the same marker analysis is applied. Finally, identifications from the initial group and the subclusters are compared and merged. For the first pass, the initial 23 clusters found by FindClusters with a resolution of 0.5 are used. First, clusters were characterized by analyzing unique marker genes for each cluster using the FindAllMarkers function with MAST set as the differential expression tool and the number of transcripts set as a variable to request as recommended by MAST. Only positive markers, i.e. genes that are uniquely upregulated in each cell type since we are interested in cell type markers and not differential expression at this stage. For each cluster, the top marker genes from FindAllMarkers were inspected via the FeaturePlot and VlnPlot functions. Each marker was then cross-referenced with the marker databases CellMarker and PanglaoDB. For each cluster, an initial identification was given based on the cell type that is canonically associated with the top markers. For example, clusters with CD4 and CD8 expression were labeled as CD4+ and CD8+ T cells respectively. Furthermore, additional cell type specific markers found within each cluster’s top markers were used to fine tune the initial identification. For example, CD4+ clusters with Il2RA or LAG3 as a top marker were labeled as regulatory T cells.

A second round of clustering was computed using FindClusters with a resolution setting of 1 which forces the clustering algorithm to generate additional subclusters, resulting in 30 total clusters. If the smaller clusters formed in this round were separate and distinct on UMAP visualization, they were included as a new cluster, overriding the cluster assignment of cells from the first round of clustering. After inspecting all clusters formed at a resolution setting of 1 and incorporating well defined clusters, some clusters were left with fewer than 300 cells total which we labeled as “orphan” clusters. All cells from these clusters were identified, then Seurat’s label transfer integration was used to reassign each individual cell to a non-orphan cluster that most closely resembles their transcriptome.

This resulted in 27 clusters, some of which already had clear identities from the first round of cell marker analysis. The remaining clusters were similarly analyzed with FindMarkers being used to find unique genes for each, and the top markers were compared against CellMarker and PanglaoDB. Some clusters remained ambivalent due to the top markers being unable to distinguish similar cell types such as naïve and memory CD8+ T cells. These clusters were further analyzed by scoring all markers against an RNA signature matrix generated from a flow cytometry sorted set of RNAseq experiments designed to generate high quality cell signatures for RNAseq deconvolution. The scoring matrix gives a score to each gene for each cell type, corresponding to its weight in determining that cell type. This scoring matrix was multiplied by the log2-fold change (logFC ) of the corresponding marker genes in the cluster in relation to the rest of the cells in the dataset, resulting in higher scores when a cluster’s marker gene has high logFC and the gene is highly weighted in the scoring matrix for a certain cell type. The top-scoring cell type for the cluster would then be assigned as the cluster cell type. After this process, two natural killer cell clusters were found to be indistinguishable and merged, resulting in a final 26 cluster dataset.

Since many of the clusters were of similar cell types that differed only by expression of specific effector genes, clusters were assigned a simplified name based on their general cell type and a numerical suffix. A broader cell type level classification was generated by removing the numeric suffixes, resulting in 15 cell type clusters.

## Cell Proportions

Cell type abundances were calculated from Seurat metadata using the standard *table* function. Percentage abundance was calculated by dividing the resulting table by its sum. This table was tested for significantly different proportions using the *pairwise.prop.test* function from the *stats* package. The test was conducted for a two-sided alternative hypothesis, with Benjamini-Hochberg correction for multiple comparisons (alternative name for the false discovery rate correction). The resulting corrected p-value tables were organized for visualization using functions from *tidyverse*. Lastly, cell proportions were plotted using *ggplot2*.

## *Differential Expression*

Differentially expressed genes were calculated from the integrated and normalized data separately in the BAL and PBMC compartments using *Seurat* and *MAST*. For each cell type, a subset of the integrated dataset was generated that only contained the cell type of interest which was then used for differential expression analysis. The *FindMarkers* function in *Seurat* normally provides a convenient wrapper for running differential expression on Seurat formatted data with MAST. However, it only supports comparison of two conditions. To make the output format more readily accessible for other functions, the FindMarkers function was modified so that when it is given three conditions, it will automatically generate hurdle models representing all three pairs of possible comparisons between groups and output differential expression data for all comparisons. All other function behaviors were left unmodified. The UMI count of each cell was used as a latent variable for regression as part of the hurdle models generated in MAST. Initial differential expression results where inspected using the *EnhancedVolcano* package.

The number of times each differentially expressed gene was detected across all cell types was computed by summing the gene names from each individual cell type’s DEG table. The data was filtered to only consider the top 17% of all DEGs. This proportion was reached by only considering genes in the PBMC compartment with adjusted p-values less than 0.05 and genes in the BAL compartment with adjusted p-values less than 1e^-7^. Next, only genes differentially expressed in at least 5 of the 15 cell types were retained after the other filtering steps. These filtering and data organizing operations were completed using *tidyverse*. Finally, per-gene variability scores generated as part of *sctransform* were extracted from the Seurat object. This was visualized with the *VariableFeaturePlot* function in Seurat; then the list of variable genes was sorted by variance from highest to lowest using *tidyverse.* The sorted set was plotted with R’s base plotting function to find the elbow where the decrease in variance from one gene to the next began to decrease. The 21 most variant genes which were plotted before this elbow point were removed using *tidyverse*. The resulting DEGs which we termed recurrent DEGs (rDEGs) are used for additional analysis.

The data was then converted to a format compatible with *Monocle 3* by extracting the normalized data and metadata from the main Seurat object used for analysis. Then, the *new_cell_data_set* function from *Monocle 3* was used to create a new CellDataSet object for module generation. The dimension reduction data from Seurat was transferred to the new CellDataSet object to maintain cluster level consistency. This dataset was subset to include only rDEGs for module generation. Then the *find_gene_modules* function in Monocle 3 was used to generate modules from the rDEGs with 30 principal components, resolution of 0.8, 100 generator iterations, and k value of 14. The *aggregate_gene_expression* function in Monocle 3 was used to generate module-level expression data of the modules, and the *pheatmap* package was used to visualize module expression levels between patient groups. The four modules were tested for differential expression between patient conditions using ANOVA testing with module expression as the dependent variable and patient conditions as the independent variable. Tukey post-hoc tests for multiple comparisons were applied to the ANOVA results to generate corrected p-values. These statistics were implemented via the base *stats* package. Module level gene ontology (GO) analysis was conducted using the *TopGO* package. A TopGO data reference was first generated from the *org.Hs.eg.db* reference package. Then the *runTest* and *GenTable* functions in TopGO were used to generate module-level GO term enrichment tables.

## Validation

Both validation datasets were imported directly to Seurat since they were available as Seurat formatted objects instead of raw count matrices. Each dataset was processed separately via the same parameters as the main dataset. First, the data was filtered to remove cells with more than 10% of transcripts associated with mitochondria genes, cells with less than 1000 UMIs, and cells with less than 200 genes detected or more than 6000 genes detected. The main integrated dataset and the validation datasets were normalized and scaled independently via SCT. Next the FindTransferAnchors and TransferData functions in Seurat were used to project and transfer our cell type identifications to the validation data. Then AddMetaData was used to transfer those predictions into the validation dataset Seurat object. Differential expression was conducted in an identical fashion to the main analysis. Our modified 3-sample differential expression function was used to find DEGs between control, mild and severe cases in each validation dataset using MAST to regress out differences in UMI count during DEG analysis. Tidyverse was then used to extract DEGs from each cell type and compare the trends against our own data, generating a new table that tracks whether findings between datasets agreed on the direction of differential expression as well as whether both found evidence of differential expression in the same cell type and gene of interest.
